# Variable use of modern abdominal wall closure techniques at emergency laparotomy – an international, cross-sectional survey of surgical practice

**DOI:** 10.1007/s00068-025-02804-y

**Published:** 2025-03-18

**Authors:** Ian J. B. Stephens, Emily Kelly, Fernando Ferreira, Marja A. Boermeester, Michael E. Sugrue

**Affiliations:** 1 Royal College of Surgeons Ireland, 123 St. Stephen’s Green, Dublin 2, Galway, Ireland; 2 School of Medicine, College of Medicine, Nursing & Health Sciences, University of Galway, Galway, Ireland; 3 Donegal Clinical Research Academy, Letterkenny University Hospital, Letterkenny, Ireland; 4 Gastrointestinal Surgery and Complex Abdominal Wall Unit, Faculty of Medicine, Hospital CUF Porto, The Oporto University, Porto, 4200-319 Portugal; 5 Amsterdam UMC, Department of Surgery, University of Amsterdam, Amsterdam, The Netherlands; 6 Department of Surgery, Letterkenny University Hospital, Letterkenny, Ireland

**Keywords:** Small bite, Laparotomy, Abdominal wall closure, Prophylactic mesh augmentation, Survey

## Abstract

**Purpose:**

Incisional hernias (IH) occur after 20–30% of laparotomies. Modern closure techniques including small bite closure and prophylactic mesh augmentation (PMA) demonstrate significant reduction in IH rates. European and American Hernia Society guidelines suggest use of small bite closure and consideration of PMA at elective laparotomy closure but do not make a recommendation for emergency surgery. International surveys demonstrate poor uptake of small bite closure and PMA. This survey aims to assess the uptake of these techniques specifically in emergency abdominal surgery.

**Methods:**

An online, cross-sectional survey was circulated through emergency general surgery (EGS) and abdominal closure networks between June and August 2024. This interrogated surgeons’ technical approach to all elements of emergency laparotomy closure including use of wound bundles, small bite, suture-to-wound ratio, suture choice, and PMA.

**Results:**

The survey was completed by 234 general surgeons from 32 countries. Wound bundle components varied between surgeons. Small bite closure was used by 85.8% during midline laparotomy closure but only 42.2% of surgeons used 5 mm wide tissue bites placed every 5 mm. Suture-to-wound ratio was rarely measured (7.7%). A looped PDS (size 0 or 1) was used preferentially (42.7%). Self-locking (15.8%) and antiseptic coated sutures (20.2%) were used infrequently. One in ten surgeons used PMA and most often placed the mesh in the retrorectus space (39.6%).

**Conclusion:**

Uptake of new techniques in emergency laparotomy has been variable and with limited penetrance amongst emergency general surgeons. Many surgeons are using adapted versions of the original descriptions of these approaches.

**Supplementary Information:**

The online version contains supplementary material available at 10.1007/s00068-025-02804-y.

## Introduction

Despite advances in minimally invasive surgery (MIS), the midline laparotomy remains the workhorse incision for abdominal access with more than 4 million laparotomies performed per annum in the United States [1]. In emergency general surgical (EGS) practice, the use of MIS approaches is limited by pathology and acuity, patient factors such as perioperative physiology and co-morbidity, and technical factors such as surgeon MIS expertise. The estimated incidence of incisional hernia (IH) after laparotomy is 20% at 2-years, and as high as 30–40% in high-risk groups such as the elderly, obese or those undergoing emergency surgery [2–4]. As IHs typically occur between 1 and 2 years after surgery, the true rate is likely underestimated both by individual surgeons in their own practice, and in the wider literature [5]. These hernias cause significant long-term morbidity and are often technically challenging to address surgically as demonstrated by the high rates of recurrence (30–40%) and re-intervention [6].

The last decade has seen randomised controlled trials (RCT) that have challenged the traditional dogma of mass closure of the abdominal wall with reduced rates of IH. “Small bite” closure of the fascia was originally described using a continuous USP size 2/0 PDS suture on a 31 mm needle, taking 5 mm tissue bites spaced 5 mm apart, incorporating only the aponeurosis and avoiding inclusion of fat or muscle. This approach demonstrated a significant reduction in IH rates at 1-year of compared to mass closure (13% vs. 21%) [7]. These findings have since been supported by further observational studies, RCTs and meta-analyses [8–11]. Similarly, prophylactic mesh augmentation (PMA) at time of laparotomy closure has demonstrated significant reductions in IH rates with both onlay (relative risk (RR) 0.24–0.26, *p* = 0.05) and retromuscular mesh (RR 0.28–0.32, *p* = 0.02) placement showing promising improvements and safety profiles compared to primary suture closure alone [6, 12]. However, these studies have been performed almost exclusively in elective or mixed elective-emergency surgery patient cohorts which limits the generalisability of these findings to high-risk subgroups such as emergency laparotomy and obese patients. While the most recent European and American Hernia Society guidelines suggest the use of small bite closure and state that PMA can be considered at elective laparotomy closure, they conclude that the limited and heterogenous nature of the data precludes a recommendation on their application to the emergency setting. Furthermore, they highlight this as a significant knowledge gap in need of further research [13].

Recent surveys of UK, Dutch and Canadian surgeons that investigated uptake of and attitudes towards these new approaches to laparotomy closure demonstrated poor uptake of small bite closure (< 10-26.7%) and PMA (3.0%) [5, 14, 15]. To date, no studies have looked specifically at international uptake of these techniques in the setting of emergency abdominal surgery. This survey aimed to address this important knowledge gap to inform future research into the subject.

## Materials and methods

Between June and August 2024, a cross-sectional, 20-question survey titled “Emergency Laparotomy Surgical Practices” was circulated internationally to general surgeons through the World Society of Emergency Surgery, Fascial Traction International, and Portuguese Surgical Society networks. This was completed in accordance with CROSS reporting guidelines for survey studies [16] (Supplemental Material 1). The questions fell into three domains– (1) surgeon demographics, (2) technical approach to emergency laparotomy closure, and (3) prevention and management of incisional hernias. The questions were designed to interrogate international practice with regards to the technical elements of abdominal wall closure including use of wound bundles, small bite-closure, and prophylactic mesh placement. Wound bundles are a package of pre-operative, intra-operative and post-operative elements designed to reduce surgical site occurrences. The exact components differ between institutes. This survey focused on the technical aspects of fascial closure but asked participants whether their local wound bundle included timed and dosed adjusted prophylactic antibiotics, skin preparation with chlorhexidine and alcohol solution, a wound protector, closure tray, and new gowns, gloves and drapes. Particular attention was paid to the specific details of fascial clearance, suture-to-wound ratio, suture choice, suture spacing, use and type of wound irrigation, and mesh type and location. The 20-questions are included in Supplemental Material 2.

The survey was administrated online, and outcome data was collected using Survey Monkey^®^ [17]. The survey was pre-tested on three European consultant general surgeons from different countries, each with a special interest in EGS. No research ethics approval was required for this survey of clinical practice. No personal information was collected from survey participants. Survey results were stored electronically in a password protected database, accessible only by the lead investigators.

Answers were collated and comments categorised. Results are reported as frequencies and percentage of total answers for a given question, excluding skipped answers. Preliminary analysis was completed using the SurveyMonkey^®^ platform, and results were exported into Microsoft Excel and STATA18^®^ [18] for statistical analysis and graphing.

## Results

A total of 234 general surgeons from 32 countries across five continents responded to the survey. Respondents were most commonly from Europe (80.7%) or Asia (10.6%), with a small number from Australia (3.9%), South (2.4%) and North (1.4%) America, New Zealand (0.5%) and Africa (0.5%). Most surgeons (45%) had been in practice for over 15 years, with almost equal numbers practicing for less than 5 years (16%), 5 to 9 years (19.9%) or 10 to 15 years (19%). All but 4 respondents (98.3%) partook in emergency abdominal surgery.

**Fig. 1:**
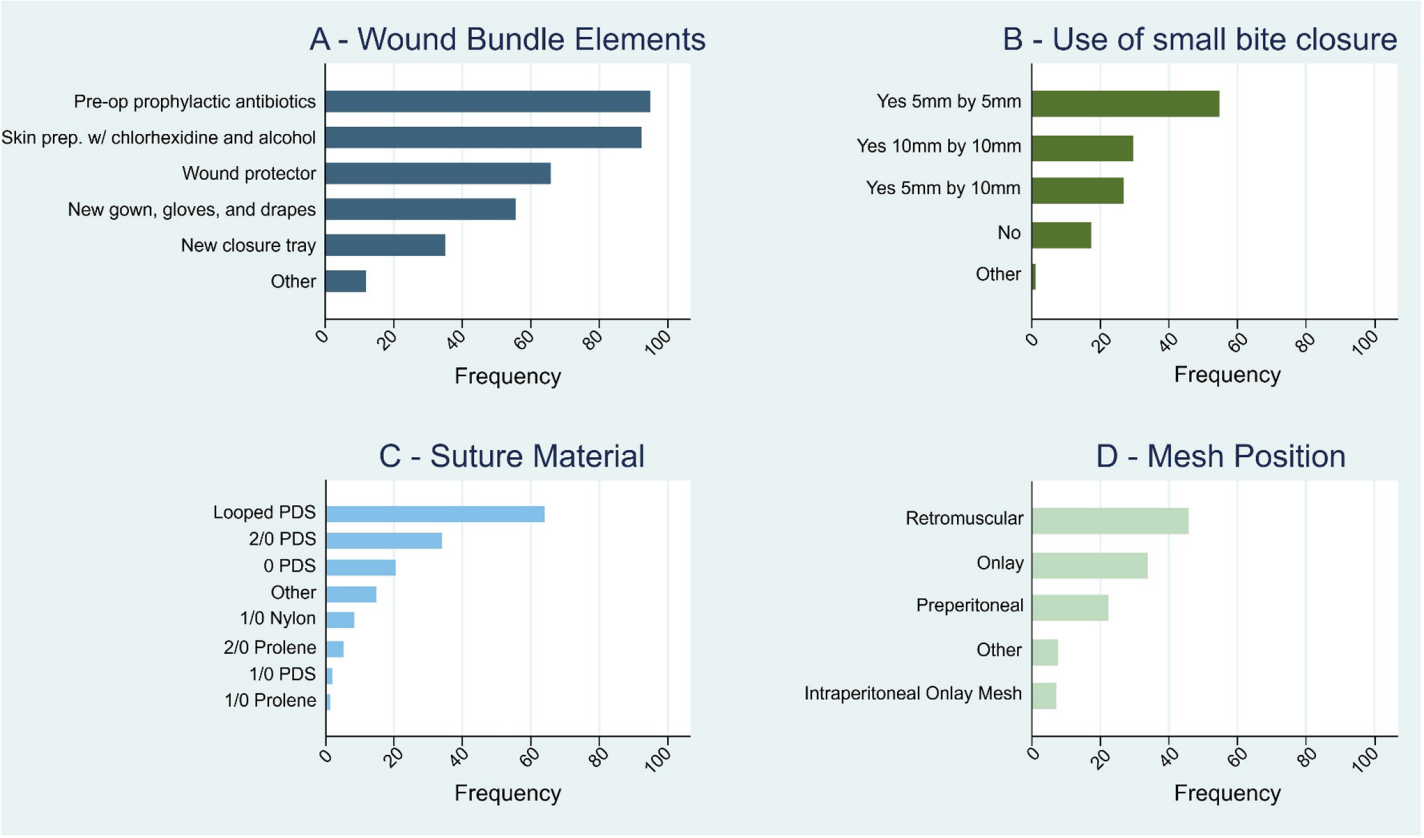
Distribution of answers to **A**) Question “wound bundle elements”, **B**) Question ‘’small bites”, **C**) Question “suture material” and **D**) Question "mesh position when using PMA". X-axis is the frequency of the response as a percentage of total survey respondents. In **B**) the width of tissue bites in mm is given first, followed by the separation between bites

Wound bundle components at time of laparotomy closure varied between surgeons. Timed dosing of pre-operative antibiotics (96.1%), skin preparation with chlorhexidine and alcohol solution (93.5%), and wound protectors (66.7%) were commonly employed. New gowns, gloves and drapes before closure were used by over half of respondents (56.3%), but new closure instruments were less common (35.5%) (Fig. 1A). Small bite closure was used by most during midline laparotomy closure (85.8%). However, the originally described 5 mm wide bites placed every 5 mm were only used by 42.2% of surgeons, with the remainder preferring different suture placements (Fig. 1B). Just over a third (37.1%) cleared 2 cm or more of the fascia of fat prior to closure. Suture-to-wound ratio was rarely measured with a ruler (7.7%). A looped PDS (size 0 or 1) was the preferred suture for closure (42.7%), followed by 2/0 PDS (22.7%) (Fig. 1C). Self-locking (15.8%) and antiseptic coated sutures (20.2%) were used infrequently. Over a third of surgeons (38.7%) used prophylactic wound irrigation. Those that did used either antibiotic containing solution, antiseptic solution, hydrogen peroxide, normal saline, Ringer’s Lactate or Hartmann’s solution. Almost two thirds (60.7%) of surgeons closed the subcutaneous space, most commonly with sutures rather than glue. Staples were the preferred means of skin closure (60.5%) compared to subcuticular suture (20.6%), or traditional interrupted suture closure. Incisional negative pressure therapy was selectively used by 68.7% of surgeons and less commonly routinely (15%) or never used (16.3%).

PMA at fascial closure was used by 1 in 10 respondents (10.7%). This was most often placed in the retrorectus space (39.6%) or in an onlay (29.2%) or preperitoneal (19.3%) position (Fig. 1D). At time of elective repair of incisional hernia, most surgeons elected for a synthetic mesh (84.2%). The retrorectus space was equally likely to be used for emergency incisional hernia repair as at PMA (45.5%). A subcutaneous drain was used selectively by most (73.8%) after mesh placement.

## Discussion

This international survey suggests a high up take of small bite closure (86.6%) in high-risk EGS patients with limited implementation of PMA (10.7%) amongst general surgeons. There was significant variability in what participants considered to constitute small bite closure and in approach to placement and type of mesh used for prevention of IH as well as wound bundle elements in use.

Compared to prior surveys of abdominal wall closure methods, we report a significantly higher (86.6%) rate of small bite closure for laparotomy closure. A recent study shows that 19.9% of surveyed NHS surgeons are using small bite; similarly a survey of Dutch surgeons suggests that over 90% still use mass closure techniques [5, 14]. Our present survey gives further granularity to the constitutive elements of the small bite approach. Though almost 90% of surgeons reported using small bite, less than half (48.8%) used the described 5 mm tissue bites with 5 mm of separation, and 26.4% used 10 mm by 10 mm bites which deviates significantly from the described technique. Equally interesting was the choice of suture, 43.9% used a looped PDS suture, which is typically either a 1 or 0 sized monofilament with a large gauge 48–65 mm needle, and less than a quarter (23.2%) used a 2/0 PDS. While biomechanical ex-vivo studies have suggested that the 5 mm by 5 mm spacing may not be the optimum for abdominal wall biomechanics, the clinical studies to date have used this configuration with significant clinical benefit [9, 19].

Uptake of PMA has been more cautious. Though multiple observational studies and clinical trials demonstrate significant reductions in IH rates with prophylactic mesh augmentation [20–25], the surgical community has ongoing reservations about this approach, particularly in the setting of peritonitis [14]. Concerns centre on fears of adverse events such as surgical site infections (SSI), chronic pain, mesh infection, mesh extrusion and enterocutaneous fistula formation [26–29]. The challenge for clinical trial design is that the most serious, traumatic, and costly of these such as mesh extrusion and enterocutaneous fistulation are rare events. It is unlikely that a real-world trial of surgical technique will be able to recruit sufficient patient numbers to reach the required statistical power to robustly compare rates of these complications between treatment arms. As such, data for these events will likely come from long-term retrospective databases and cohorts which will take time to materialise and are subject to multiple biases.

Wound bundles, which incorporate multiple, typically small scale or lower impact, perioperative elements have shown promise in reducing rates of surgical site occurrences including SSI and IH [30–32]. This survey has demonstrated variability between surgeons in the use of elements such as wound protectors, change of gloves and instruments for closing, wound irrigation, use of incisional negative pressure therapy, more ubiquitous use of pre-operative antibiotics and skin preparation with chlorhexidine and alcohol. While some elements of a bundle such as small bite closure [9], PMA [24, 27], pre-operative antibiotics, and antiseptic skin [33] preparation provide large impact on outcome measurements that are evident in small to medium scale RCTs. Others such as change of gloves and instruments [34] and wound irrigation [33, 35–38] may have much smaller effect size but their benefits have been demonstrated. High level evidence shows that wound irrigation with antiseptic solutions such as aqueous povidone iodine effectively reduce SSI [35], while polyhexanide solution is ineffective [33]. Furthermore, precise details such as antiseptic concentration can have significant impacts on outcomes as demonstrated by a recent network meta-analysis indicating that chlorhexidine 2-2.5% in alcohol is superior to other skin preparation solutions in the prevention of SSIs [33]. The flip-side of this is that the potential risks of implementation vary significantly between elements from the high-impact such as abdominal dehiscence or enterocutaneous fistula to the low-impact such as dermatitis. As a consequence, a tug-of-war exists in the uptake of such approaches in the surgical community whereby the balance shifts between the perceived beneficial outcomes for the majority relative to the often rare but highly significant harms to the minority. The apparent use of “augmented” small bite closure such as 5 mm by 5 mm bites but with large diameter blunt needles and sutures, demonstrated by this survey speaks to the fear we as surgeons carry when it comes to implementing changes in our practice that differ drastically from what we are taught in our training. Unfortunately, such variable implementation of techniques as they are more widely adopted and altered compared to their original descriptions likely dilutes the impact on outcomes for subsequent retrospective analyses. It is essential that surgical units regularly audit their own practice and know their own short- and long-term complication rates to allow for the successful implementation of changes in practice.

### Limitations

There are several limitations to this survey. The participants were recruited from the World Society of Emergency Surgery, Portuguese Surgical Society, and Fascial Traction International which would suggest an interest and scholarship in abdominal wall closure and EGS. This may have introduced a bias in favour of small bite closure and PMA compared to the overall general surgical community. This is supported by the results, which suggest over three times the uptake of small bite closure and PMA compared to prior surveys, despite specifically addressing emergency laparotomy. A further limitation is the lack of specific details relating to wound bundle elements which were addressed in a single broad ranging question, despite varying significantly in implementation between institutions. Finally, the questions did not address barriers to uptake or change in practice. It is not possible to conclude whether this was limited by knowledge or practical constraints such as hands on experience, operating theatre time, case contamination or patient factors.

## Conclusions

Even amongst surgeons with a declared interest in emergency general surgery and abdominal wall closure, uptake of new techniques such as wound bundles, small bite closure, and prophylactic mesh augmentation in emergency laparotomy has been variable and with limited penetrance. The patterns of uptake suggest that surgeons are using technical variations of the original descriptions of these approaches. There is a clear need for further RCTs specifically investigating the use of these approaches in the emergency general surgery patient cohort to reduce variability, and more clearly define optimal surgical approaches.

## Electronic supplementary material

Below is the link to the electronic supplementary material.

Supplementary Material 1

## Data Availability

Data will be provided by authors upon reasonable request.
